# A New Destination on the Palm? The Moderating Effect of Travel Anxiety on Digital Tourism Behavior in Extended UTAUT2 and TTF Models

**DOI:** 10.3389/fpsyg.2022.965655

**Published:** 2022-07-27

**Authors:** Jiaojiao Sun, Yingzhi Guo

**Affiliations:** ^1^School of Business, Suzhou University of Science and Technology, Suzhou, China; ^2^Postdoctoral Station of Business Administration, Fudan University, Shanghai, China

**Keywords:** digital tourism, UTAUT2 model, TTF model under social distancing, pandemic anxiety travel scale, use intentions, use behavior

## Abstract

Digital tourism has developed rapidly, especially in museums. However, as people become increasingly familiar with digital museums, their use intentions and behavior have changed. Taking the Digital Palace Museum in China as an example, applying the PLS-SEM method, this study uncovers visitors’ use intentions and actual use behavior for digital museums by integrating the new UTAUT model (UTAUT2) and TTF model (TTF under social distancing) and introduces the PATS (Pandemic Anxiety Travel Scale) model to reveal how pandemic anxiety promotes the transformation of use intentions into use behavior more easily. The results show that performance expectations, hedonic motivations, habits, and task-technology-fit positively affect use intentions for digital museums. However, the price-saving orientation negatively affects use intentions. Pandemic anxiety moderates the effect of use intentions on actual behavior. When travel anxiety is relatively high, use intentions have a greater effect on use behavior for digital museums. The results reveal the influencing factors on use intentions of digital museums and the moderating effect of pandemic anxiety on the relation between use intentions and actual behavior.

## Introduction

Digital tourism is a typical manifestation of the tourism industry’s paradigm shifts (Joo et al., 2021). During the past few years, tourists’ risk perceptions (Karl et al., 2020; Rather, 2021) created great obstacles to people’s tourism behavior and the tourism industry was severely affected (Rasoolimanesh et al., 2021). Under this background, technology-based tourism activities have become one of the most critical alternatives to overcome these obstacles (Itani and Hollebeek, 2021; Lv et al., 2022) since Internet technologies facilitate safe distancing in tourism activities (García-Milon et al., 2021; Nanni and Ulqinaku, 2021). As a result, tourism destinations rapidly began promoting digital virtual tourism experiences online (Talafubieke et al., 2021; Zhang et al., 2022) to capture tourist revenue by applying technologies such as online applications and digital instruments (Bec et al., 2021).

As an important component of tourist destinations (Jin et al., 2020), the museums in China are facing a key problem of over-crowding (Chen and Ryan, 2020). To tackle this issue, the digital transformation with the application of virtual (VR) and augmented reality (AR) technologies (Zhang et al., 2022) has become an effective way out. Researchers have previously analyzed the effect of digital technologies on visitors’ purchase intentions (e.g., He et al., 2018). However, the new value of digital museums is in their role as an alternative experience to on-site presence. Museums are currently experiencing new challenges and adjustments in the transformation of their customer orientation from “high-touch” to “high-tech” (Wang et al., 2021).

What is more challenging is that consumers have frequently interacted with high-tech service (Yang et al., 2022) and become increasingly familiar with digital tourism; therefore, their digital product needs and requirements change constantly. However, online tourism is still in its infancy for many tourism destinations (Chen et al., 2021). Therefore, it is still difficult for researchers to understand the public’s attitude toward virtual tourism during a crisis (Zhang et al., 2022), such as the travel restrictions caused by the epidemic. Both practical and theoretical fields have raised questions for the new research field, such as what elements of online tourism products determine people’s use intentions and behavior?

Traditional studies have used the UTAUT model (unified theory of acceptance and use of technology) and the TTF model (task–technology fit) to analyze customers’ acceptance of science and technologies. However, most studies consider the use of these technologies as supporting tourists’ offline behavior, such as making their travel plans more convenient through digital payment or online reservation methods (Martín and Herrero, 2012; Escobar-Rodríguez and Carvajal-Trujillo, 2014; Wu et al., 2021; Erjavec and Manfreda, 2022). Nevertheless, the pandemic accelerates the wide application of Internet tools (García-Milon et al., 2021) and many tourism destinations have created digital platforms to replace their on-site experiences with digital experiences. In contrast with auxiliary functions such as online payments and reservations, digital experiences replace the original on-site experience, and studies of consumer responses to these new initiatives are still scant (Itani and Hollebeek, 2021). Therefore, the variables used in the UTAUT and TTF models should be reconsidered in this new situation. However, few studies have researched this aspect of online tourism. Although scholars have discussed the use intentions for digital platforms, it is still unclear how these use intentions could transform into use behavior.

Three research gaps were identified:

*Gap 1*: When people become increasingly familiar with digital platforms, their requirements for digital experiences increase. Therefore, it is necessary to update and integrate the original model variables to better explain use intentions and behavior for digital museums.
*Gap 2*: The combination of updated UTAUT (from the consumer perspective) and TTF (from the supplier perspective) models is required to explain the use of digital museums to substitute for on-site tourism activities, which differs from earlier studies that considered digital platforms as a subsidiary tool for on-site tourism.
*Gap 3*: Can the travel anxiety influence the transformation of digital museum use intentions into actual use behavior?

Therefore, researchers are encouraged to analyze digital tourism experiences. The UTAUT and TTF models have great explanatory power in traditional studies of digital behavior for online businesses because they can predict use intentions from the perspectives of supplier technologies and user experiences. However, the UTAUT and TTF models should also be considered in the context of a pandemic situation.

Based on the above, taking digital museums as an example, using data collected by the questionnaire survey, and applying the PLS-SEM method, this study integrates the new UTAUT model (UTAUT2) and TTF model (TTF under social distancing), introduces the scale of pandemic anxiety, explores how the different characteristics of online products and people’s personal needs affect their use intentions for digital museums and how the pandemic anxiety promotes or hinders the transformation of use intentions into use behavior.

## Literature Review

### The Original and Updated TTF Models

Goodhue (1998) developed the TTF model to explain the relationship between technology and consumers’ acceptance, performance (Hung et al., 2021), and use (Kim et al., 2010). The TTF model refers to the characteristics of technologies and tasks, and how they fit together. In addition, the TTF model can also be explained as matching technological capabilities to task demands (Kim et al., 2015) or as the extent to which technological tools can help individuals to complete their tasks (Hung et al., 2021). The TTF model is used widely in analyzing use intentions for digital libraries, health care, and decision-making, in addition to the effects on performance, attitudes, and beliefs (McGill and Klobas, 2009).

Many leisure and learning interactions were digitalized during the pandemic; thus, consumers have embraced a variety of digital forms (Wang et al., 2021). The TTF model is an important model for studies of the effect of online experiences, such as technology-facilitated student learning activities (Lçi and Abubakar, 2021). However, the characteristics of the two main factors in the TTF model, technologies and tasks, have changed during the past 2 years of the pandemic (Wu et al., 2021). On the one hand, consumers’ technology demands are increasing. On the other hand, the change in task characteristics is reflected in the influence of offline social distancing. Therefore, Wang et al. (2021) also upgraded the specifically related concepts: namely, the characteristics of tasks (TAS) and technologies (TEC) under social distancing. In addition, Mehraliyev et al. (2020) demonstrated the effect of TTF on use intentions. Thus, we propose H1a, H1b, and H1c:

*H1a*: Characteristics of tasks under social distancing (TAS) positively affect TTF.

*H1b*: Characteristics of technologies under social distancing (TEC) positively affect TTF.

*H1c*: The TTF model positively affects use intentions for digital museums.

### The UTAUT and UTAUT2 Models

Venkatesh et al. (2003) constructed the UTAUT model to explain the initial and repeated adoption of technologies (Venkatesh et al., 2012; Fong et al., 2017). The UTAUT model was constructed from the factors that affect usage intentions (Escobar-Rodríguez and Carvajal-Trujillo, 2014). In their tourism study, Martín and Herrero (2012) analyzed the online purchase intention regarding rural tourism.

Four core factors anchor the traditional UTAUT model (Fong et al., 2017; Abou-Shouk and Soliman, 2021): performance expectancy (PE), effort expectancy (EE), social influence (SI), and facilitating conditions (FC). PE is an essential element in the adoption of new technologies because it refers to the degree that tourists consider that using a digital museum will be beneficial (Venkatesh et al., 2003; García-Milon et al., 2021). Tourism studies have demonstrated the positive influence of PE on acceptance and use intentions for new technologies (García-Milon et al., 2021). EE could be understood as the degree of ease (Venkatesh et al., 2003; García-Milon et al., 2021) in using digital museums. Studies have identified the positive effect of EE on behavior intentions (García-Milon et al., 2021). SI relates to the extent to which tourists believe that key individuals in their lives believe they should visit digital museums (Venkatesh et al., 2003; García-Milon et al., 2021). The positive effect of SI on behavioral intentions was demonstrated, especially in tourism activities (García-Milon et al., 2021). FC refers to the degree to which individuals consider that there is an organizational and technical structure to support them in their use intentions (Venkatesh et al., 2003; García-Milon et al., 2021). García-Milon et al. (2021) also demonstrated the positive effect of FC on use intentions for innovative technologies in tourism activities. Martín and Herrero (2012) identified the positive effects of PE, EE, SI, and FC on online purchase intentions. Based on these findings, we propose the following hypotheses:

*H2a*: Performance expectancy (PE) positively affects use intentions for digital museums.

*H2b*: Effort expectancy (EE) positively affects use intentions for digital museums.

*H2c*: Social influence (SI) positively affects use intentions for digital museums.

*H2d*: Facilitating conditions (FC) positively affects use intentions for digital museums.

Venkatesh et al. (2012) constructed the UTAUT2 model further by upgrading the earlier UTAUT model through introducing three new elements, namely hedonic motivations (HM), price value (PV) or price-saving orientation (PO), and habits (HT). The updated version of the UTAUT model is more appropriate for studying technology adoption from a consumer perspective (Escobar-Rodríguez and Carvajal-Trujillo, 2014). The positive effect of the new variable on use intentions was identified (Escobar-Rodríguez and Carvajal-Trujillo, 2014). Although PO was previously considered to be a positive factor affecting use intentions for online platforms, people’s normal consumption demands were suppressed during the pandemic, which resulted in abnormal consumption demand and consumption psychology. Scholars subsequently identified the new behavioral trends of “retaliation consumption” (Qiao et al., 2021) and “compensatory consumption” (He et al., 2021). People confined to their homes by the pandemic are in urgent need of psychological satisfaction (Cheung et al., 2021) through online consumption, such as the digital economy consumption demands of online content like games (Xu et al., 2016), reading, and watching videos (Zheng, 2020). Hence, consumers will participate in digital museum experiences to facilitate their consumption behavior instead of in attempt to save costs. Therefore, this study assumes a new effect of PO on use intentions for digital museums. Thus, we propose hypotheses of H3a, H3b, H3c, and H4:

*H3a*: Hedonic motivations (HM) positively affects use intentions for digital museums.

*H3b*: Price-saving orientation (PO) negatively affects use intentions for digital museums.

*H3c*: Habits (HT) positively affects use intentions for digital museums.

*H4*: Use intentions positively affect use behavior for digital museums.

### Pandemic Anxiety and Digital Travel Behavior

Many concepts have been established to describe the effect of the pandemic on travel behavior, such as travel fears and health concerns (Shin et al., 2022), which lead to protection motivation and travel avoidance (Zheng et al., 2021). Zenker et al. (2021) constructed the PATS model, namely the pandemic anxiety travel scale to measure people’s travel anxiety and found that pandemic anxiety harmed their intentions to travel.

**Figure 1:**
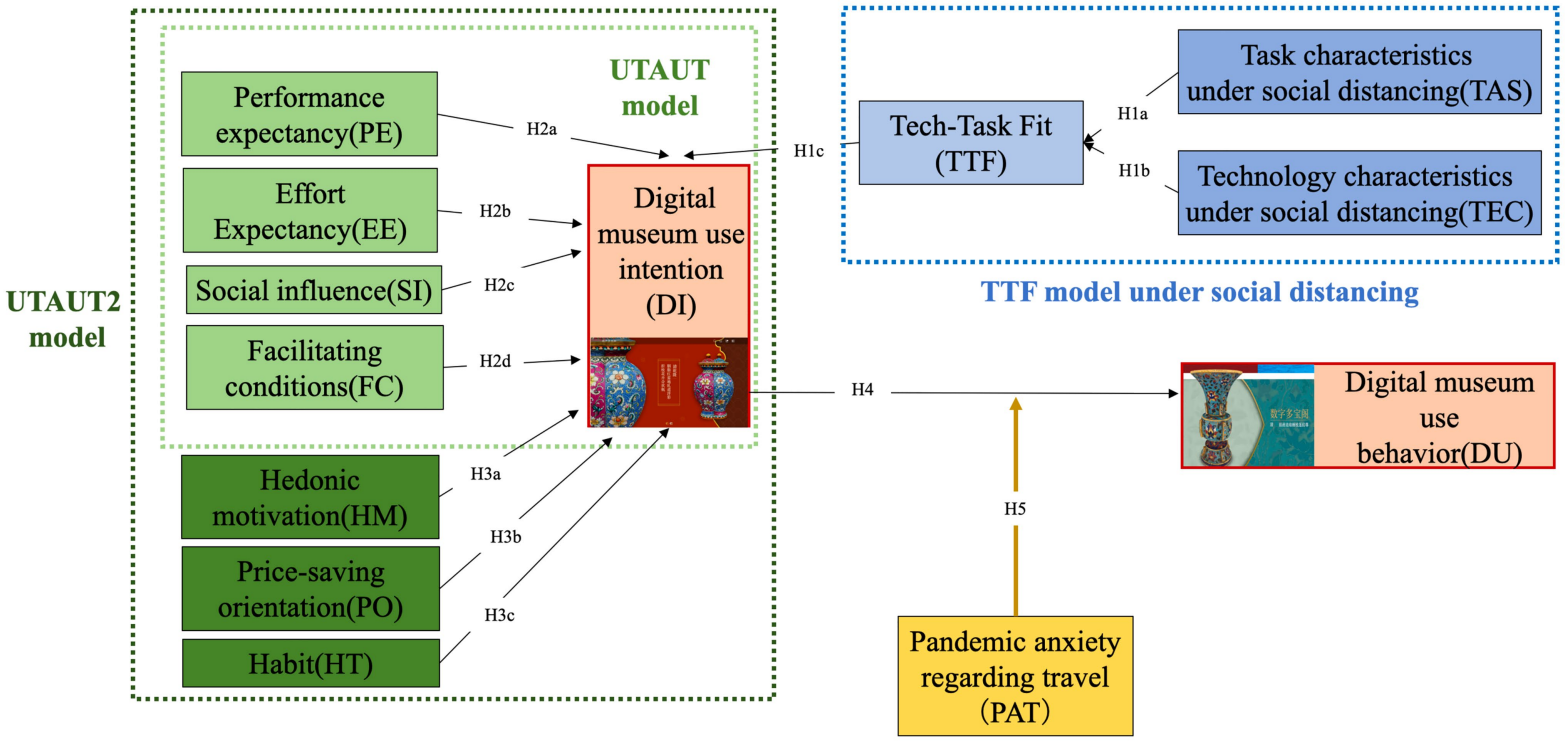
Conceptual model.

Travel fear evokes different coping strategies (Zheng et al., 2021; Zhang et al., 2022) and perceived risk is linked to engagement in protective behaviors (Villacé-Molinero et al., 2021). Therefore, digital experiences have become an important alternative. Studies have identified tourists’ positive sentiments about virtual tourism (Zhang et al., 2022) and their potential switch to technologically safe substitutes (Nanni and Ulqinaku, 2021). In addition, García-Milon et al. (2021) identified the moderating role of the pandemic anxiety on the willingness of using smartphones in tourism experiences. Considering digital behavior as a substitute for tourism behavior in visiting museums, will the pandemic anxiety affect the use behavior for digital museums? Thus, we propose the following hypothesis and show our research model in Figure 1.

*H5a*: Pandemic anxiety regarding travel positively moderates the effect of use intentions on use behavior for digital museums.

## Methodology

### Research Context

**Figure 2:**
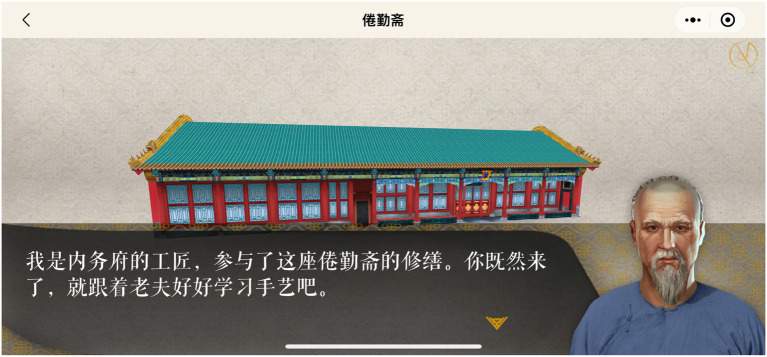
DPM: guided VR experience (the texts on the top of the figure show the name of the building “Qinjuanzhai”; the role in the figure says: I am a craftsman of the Imperial Household Department and participated in the repair of the House of Qinjuanzhai. Since you are here, you can learn the craft well with me).

**Figure 3:**
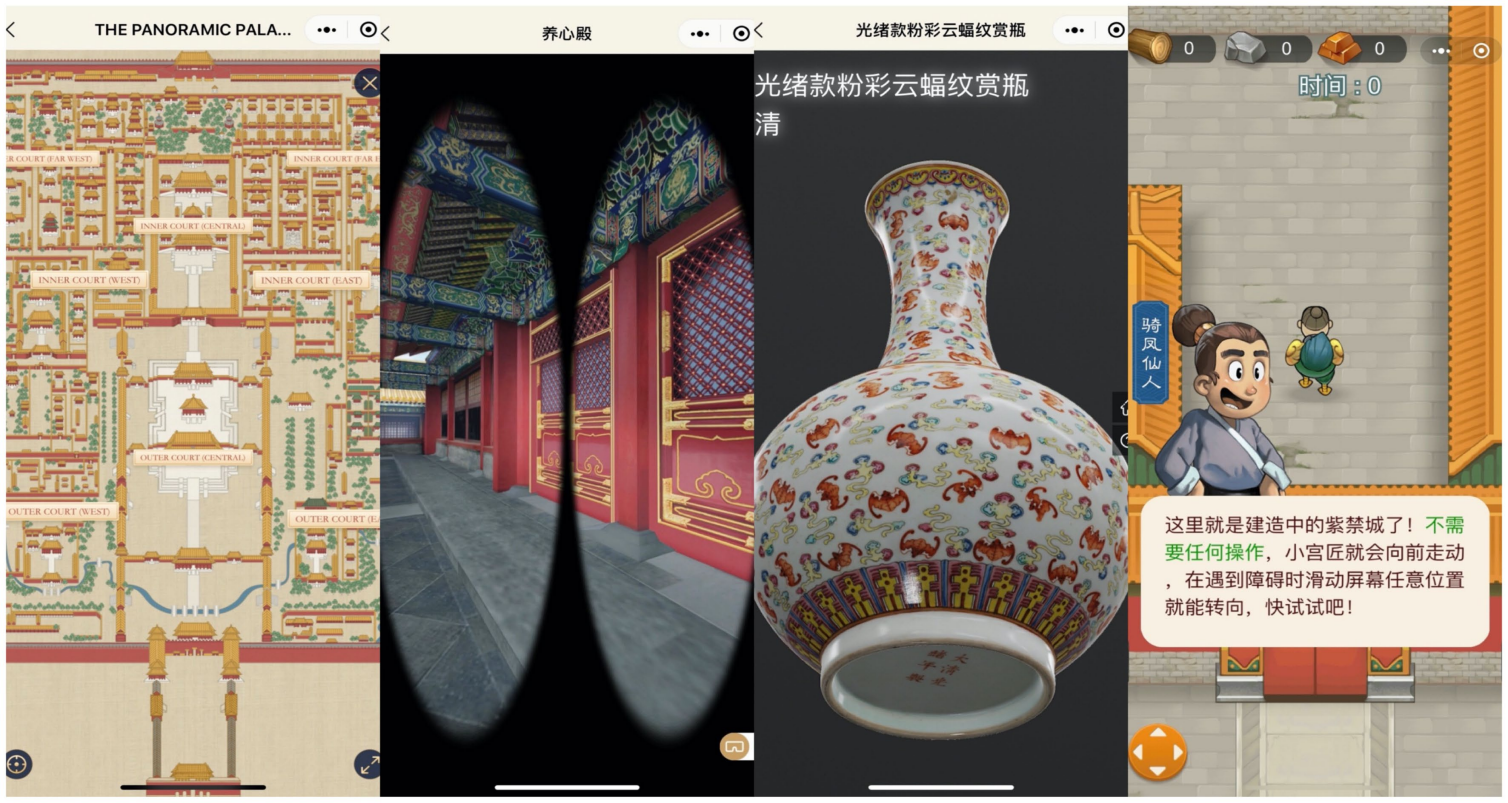
DPM: 3D map, VR tours, digital relics, and virtual tour games (the texts above the vase show the name of the vase and the dynasty of Qing, the cartoon character says: This is the Forbidden City under construction! Without any operation, the craftsman will move forward. When encountering obstacles, you can swipe the screen to make him turn. Try it!).

Located in the heart of Beijing, China, the Palace Museum is a World Cultural Heritage Site. The Palace Museum is based on the Ming and Qing dynasties’ imperial palaces, artworks and collections. In July 2020, the Palace Museum and Tencent jointly created a WeChat mini program called Digital Palace Museum (DPM), which is an applet based on the WeChat app. With more than 1.26 billion daily users (Tencent, 2022), WeChat has become the most widely used social media application in China. Within 1 year since its launch, the number of users of Digital Palace Museum applet has exceeded 5.5 million, and the number of visits has exceeded 23 million (Guangming Net, 2021). On 21 December 2021, the DPM applet was officially updated to version 2.0, which introduced multiple new functions, such as AR live navigation, location-based services (LBS), a built-in “customized tour route,” and a bookstore, in which souvenirs can be purchased and directly mailed home (CNBN, 2021). During the pandemic, the innovative DPM applet has become a representative form of the Palace Museum and follows digital trends by offering multiple digital experiences, such as VR experiences with guides (Figure 2), three-dimensional (3D) maps, VR tours, digital relics, and virtual tour games (Figure 3), and has changed Palace Museum visitors’ experiences and expectations.

### Data Collection and Measures

Using a questionnaire and interviews, this study collected data from people who had used the “digital Palace Museum” app. The five-part questionnaire adopted a Likert seven-point scale (1 = “strongly disagree” and 7 = “strongly agree”) and the respondents chose the corresponding scores based on their own experience. The first part of the questionnaire collects personal data, including gender and age, the second part uses the UTAUT2 model scale, the third part uses the scale of the TTF model under social distancing, the fourth part captures use intentions and use behavior for digital museums, and the fifth part is the PATS (Table 1). The sampling process consists of two steps. First, by conducting an open survey through the dominant communication apps in China, including Tencent QQ, WeChat, and Weibo, in addition to face-to-face in person, the researchers identified the potential participants who had used the “Digital Palace Museum” applet. Second, after determining the pool of potential participants, the researchers established and distributed paper and digital versions of the questionnaires from 2 January to 28 January 2022. In total, 236 questionnaires were collected and 15 were rejected, leaving 221 complete questionnaires with an effective rate of 93.44%. Table 2 shows the demographic information of the respondents.

**Table 1:** Measuring scales.

Construct	Items	Sources
Characteristics of tasks under social distancing (TAS)	TAS1: I need to experience the museum without direct contact with other people. TAS2: I need to avoid unnecessary social contact for my daily activities including experiencing the museum. TAS3: I need to experience the museum while staying at home as much as possible.	Wang et al., 2021
Characteristics of technologies under social distancing (TEC)	TEC1: These technologies help me to avoid unnecessary social contact. TEC2: These technologies help me to comply with social distancing practices. TEC3: These technologies enable me to stay at home as much as possible.	Wang et al., 2021
Task-technology fit (TTF)	FIT1: The technologies’ functions are sufficient in helping me to complete the museum experience activities. FIT2: The technologies functions are appropriate in helping me to complete the museum experience activities. FIT3: In general, the functions of these technologies fully meet my museum experience needs.	Zhou et al., 2010; Wang et al., 2021
Performance expectancy (PE)	PE1. Using the digital museum for my museum experience is useful PE2. Using the digital museum increases my chance of achieving my goals PE3. Using digital museum allows me to experience quicker PE4. Using digital museum helps me to be more productive in the experience process	Fong et al., 2017; García-Milon et al., 2021; Erjavec and Manfreda, 2022
Effort expectancy (EE)	EE1. It is easy for me to learn to use the digital museum EE2. Using the digital museum is clear and understandable EE3. It is easy for me to use the digital museum EE4. It is easy for me to be proficient in using the digital museum	Fong et al., 2017; García-Milon et al., 2021; Erjavec and Manfreda, 2022
Social influence (SI)	SI1. People who are important to me think that I should use the digital museum for my museum experience purpose SI2. People who influence me think that I should use the digital museum for my museum experience purpose SI3. People whose opinions I value would prefer I use a digital museum for my museum experience purpose	Fong et al., 2017; García-Milon et al., 2021; Erjavec and Manfreda, 2022
Facilitating conditions (FC)	FC1. I have the resources necessary to use a digital museum FC2. I have the knowledge to use a digital museum FC3. The digital museum is compatible with other technologies I use for museum experience FC4. I can get help from others if I have difficulty using the digital museum	Fong et al., 2017; García-Milon et al., 2021; Erjavec and Manfreda, 2022
Hedonic motivation (HM)	HM1. Using the digital museum websites is fun. HM2. Using the digital museum websites is enjoyable. HM3.Using the digital museum websites is very entertaining.	Escobar-Rodríguez and Carvajal-Trujillo, 2014
Price-saving orientation (PO)	PO1. I can save money by using the digital museum websites. PO2. I like to search for cheap offers on different digital museum websites. PO3. The digital museum websites offer better value for my money.	Escobar-Rodríguez and Carvajal-Trujillo, 2014
Habit (HT)	HT1. The use of the digital museum websites has become a habit for me. HT2. I am addicted to using the digital museum websites. HT3. I must use the digital museum websites.	Escobar-Rodríguez and Carvajal-Trujillo, 2014
Digital museum use intention (DI)	DI1.I intend to use the digital museum in the future. DI2.I will always try to use the digital museum in my daily life. DI3.I plan to continue to use the digital museum frequently.	Fong et al., 2017; Erjavec and Manfreda, 2022
Digital museum use behavior (DU)	DU1. How often do you use the digital museum?	Escobar-Rodríguez and Carvajal-Trujillo, 2014
Pandemic anxiety travel scale (PATS)	PATS1 COVID-19 make me worry a lot about my normal ways of travelling. PATS2 It makes me uncomfortable to think about COVID-19 while planning my vacation. PATS3 I am afraid to risk my life when I travel, because of COVID-19. PATS4 When watching news about COVID-19, I become nervous or anxious in regards to travel. PATS5 I do not feel safe to travel due to COVID-19	Zenker et al., 2021

**Table 2:** Respondents’ demographic information.

Item	Categories	Response	Percentage
Age	Under 18	23	10.41%
	18–27	68	30.77%
	28–37	58	26.24%
	38–47	50	22.62%
	48–57	18	8.14%
	58 and above	4	1.81%
Gender	Female	112	50.68%
	Male	109	49.32%
Education	Primary/middle school	15	6.79%
	High school/secondary school	52	23.53%
	junior college/ undergraduate	89	40.27%
	graduate and postgraduate	65	29.41%
Monthly Income	Under 2000 RMB	26	11.76%
	2,000–5,000 RMB	69	31.22%
	5,000–8,000 RMB	80	36.20%
	Above 8,000 RMB	46	20.81%

## Results

### Measurement Model Analysis

The analysis and estimation procedure for partial least squares–structural equation modeling (PLS-SEM) can be divided into two steps. The reliability and validity of the measurement model were analyzed and the path coefficient test and model prediction ability estimation were performed for the structural model. To evaluate the reliability and validity of PLS-SEM, we must first calculate each variables’ composite reliability (CR) and average variance extracted (AVE). The CR of potential variables and Cronbach’s α all indicate the consistency of items within the variable. When Cronbach’s α is greater than 0.7, it can be considered to have good internal consistency (Fornell and Larcker, 1981). AVE is a measure of the ability of a potential variable to explain all its index items and its value must be greater than 0.5 to prove that it is feasible. In this study, the minimum value of Cronbach’s α is 0.762 and the minimum value of CR is 0.845, both of which are greater than 0.7, indicating that the potential variables in this study have good internal consistency. The minimum AVE value of potential variables is 0.579, which is greater than 0.5, indicating that the potential variables in this study have good convergence validity (Fornell and Larcker, 1981; Table 3).

**Table 3:** Confirmatory factor analysis results.

Variables	Items	Factor loadings	Cronbach’s alpha	CR	AVE
TAS	TAS1	0.932	0.910	0.943	0.847
	TAS2	0.921			
	TAS3	0.908			
TEC	TEC1	0.951	0.937	0.960	0.889
	TEC2	0.954			
	TEC3	0.922			
TTF	TTF1	0.909	0.904	0.940	0.839
	TTF2	0.907			
	TTF3	0.933			
PE	PE1	0.793	0.867	0.909	0.715
	PE2	0.844			
	PE3	0.878			
	PE4	0.864			
EE	EE1	0.765	0.762	0.845	0.579
	EE2	0.693			
	EE3	0.862			
	EE4	0.711			
SI	SI1	0.857	0.764	0.866	0.686
	SI2	0.901			
	SI3	0.715			
FC	FC1	0.901	0.926	0.947	0.817
	FC2	0.897			
	FC3	0.925			
	FC4	0.893			
HM	HM1	0.737	0.774	0.869	0.691
	HM2	0.867			
	HM3	0.882			
PO	PO1	0.798	0.823	0.895	0.740
	PO2	0.885			
	PO3	0.894			
HT	HT1	0.920	0.902	0.939	0.836
	HT2	0.911			
	HT3	0.912			
DI	DI1	0.908	0.877	0.924	0.801
	DI2	0.909			
	DI3	0.868			
DU	1.000	1.000	1.000	1.000	1.000

The discriminant validity test can be achieved through three pathways. The first pathway is cross-loading, where the factor loadings of the items in each variable are higher than that of items in other variables (Table 4). The second pathway is Fornell and Larcker’s (1981) criterion. When the AVE value of the variable is greater than the square of the correlation coefficient between the variable and other variables, the result indicates discriminant validity. Because the AVE value is the square value of the standardized factor loading of the horizontal item under the same potential variable, the square roots are required during comparison. In this study, the AVE meets the standard (Table 5). The third pathway is the heterotrait–monotrait (HTMT) ratio test. Henseler et al. (2015) suggested that the HTMT ratio should be added to the discriminant validity analysis. When the HTMT value between two different latent variables is greater than 0.85, the result indicates that it can pass the discriminant validity test. The correlation coefficients between latent variables are 0.115 to 0.843, which meets this standard (Table 6). In summary, the validity of our findings was tested in three ways, indicating that the data passed the discriminant validity test.

**Table 4:** PLS-SEM factor loadings (bold) and cross loadings.

	TAS	TEC	TTF	PE	EE	SI	FC	HM	PO	HT	DI	DU
TAS1	**0.908**	0.610	0.557	0.634	0.452	0.325	0.217	0.264	0.312	0.310	0.435	0.464
TAS2	**0.932**	0.588	0.636	0.700	0.493	0.205	0.200	0.216	0.262	0.297	0.482	0.449
TAS3	**0.921**	0.614	0.610	0.673	0.424	0.233	0.174	0.180	0.271	0.239	0.448	0.475
TEC1	0.633	**0.951**	0.673	0.612	0.502	0.377	0.133	0.324	0.381	0.301	0.477	0.630
TEC2	0.633	**0.954**	0.694	0.631	0.489	0.393	0.121	0.338	0.347	0.315	0.507	0.624
TEC3	0.586	**0.922**	0.604	0.555	0.432	0.363	0.044	0.290	0.365	0.253	0.415	0.567
TTF1	0.620	0.735	**0.909**	0.672	0.510	0.328	0.203	0.226	0.347	0.251	0.566	0.612
TTF2	0.579	0.537	**0.907**	0.660	0.508	0.326	0.247	0.230	0.282	0.280	0.677	0.562
TTF3	0.599	0.644	**0.933**	0.678	0.549	0.340	0.178	0.238	0.321	0.268	0.651	0.625
PE1	0.672	0.536	0.588	**0.793**	0.414	0.239	0.151	0.230	0.327	0.349	0.480	0.494
PE2	0.536	0.421	0.537	**0.844**	0.483	0.263	0.210	0.242	0.357	0.348	0.544	0.454
PE3	0.643	0.561	0.630	**0.878**	0.543	0.289	0.204	0.226	0.362	0.347	0.556	0.490
PE4	0.621	0.638	0.718	**0.864**	0.590	0.409	0.108	0.314	0.386	0.316	0.552	0.633
EE1	0.519	0.545	0.477	0.579	**0.765**	0.354	0.202	0.333	0.220	0.222	0.440	0.567
EE2	0.186	0.181	0.241	0.224	**0.693**	0.155	0.224	0.241	0.148	0.187	0.206	0.164
EE3	0.427	0.416	0.552	0.554	**0.862**	0.296	0.252	0.352	0.255	0.225	0.464	0.477
EE4	0.282	0.293	0.370	0.360	**0.711**	0.173	0.248	0.214	0.223	0.296	0.396	0.319
SI1	0.196	0.206	0.246	0.281	0.275	**0.857**	0.159	0.512	0.203	0.133	0.305	0.269
SI2	0.236	0.302	0.289	0.278	0.271	**0.901**	0.178	0.539	0.286	0.199	0.288	0.293
SI3	0.249	0.499	0.369	0.328	0.296	**0.715**	0.048	0.326	0.188	0.166	0.277	0.405
FC1	0.239	0.183	0.251	0.217	0.310	0.138	**0.901**	0.190	0.309	0.442	0.251	0.271
FC2	0.128	0.015	0.144	0.116	0.170	0.121	**0.897**	0.087	0.161	0.299	0.183	0.118
FC3	0.209	0.062	0.204	0.188	0.271	0.159	**0.925**	0.185	0.236	0.323	0.212	0.198
FC4	0.177	0.103	0.209	0.186	0.317	0.148	**0.893**	0.088	0.268	0.332	0.217	0.167
HM1	0.119	0.278	0.192	0.194	0.267	0.391	0.109	**0.737**	0.037	0.143	0.290	0.268
HM2	0.214	0.207	0.204	0.274	0.359	0.484	0.122	**0.867**	0.159	0.232	0.371	0.213
HM3	0.244	0.360	0.233	0.271	0.322	0.509	0.156	**0.882**	0.288	0.351	0.375	0.288
PO1	0.271	0.244	0.274	0.322	0.254	0.138	0.343	0.145	**0.798**	0.579	0.239	0.195
PO2	0.271	0.409	0.301	0.367	0.252	0.297	0.222	0.168	**0.885**	0.637	0.194	0.288
PO3	0.246	0.354	0.315	0.401	0.235	0.278	0.148	0.211	**0.894**	0.651	0.253	0.320
HT1	0.283	0.302	0.278	0.361	0.277	0.198	0.373	0.260	0.734	**0.920**	0.307	0.241
HT2	0.282	0.296	0.254	0.351	0.297	0.174	0.385	0.293	0.648	**0.911**	0.299	0.254
HT3	0.273	0.255	0.264	0.385	0.273	0.178	0.325	0.268	0.614	**0.912**	0.358	0.229
DI1	0.484	0.423	0.632	0.562	0.426	0.266	0.302	0.354	0.221	0.299	**0.908**	0.510
DI2	0.491	0.560	0.709	0.652	0.557	0.406	0.161	0.399	0.330	0.370	**0.909**	0.613
DI3	0.331	0.315	0.474	0.456	0.403	0.249	0.191	0.368	0.146	0.267	**0.868**	0.426
DU	0.502	0.645	0.655	0.613	0.543	0.388	0.215	0.306	0.312	0.263	0.586	**1.000**

**Table 5:** Discriminant validity (intercorrelations) of constructs (Fornell and Larcker).

	TAS	TEC	TTF	PE	EE	SI	FC	HM	PO	HT	DI	DU
TAS	0.920											
TEC	0.656	0.943										
TTF	0.655	0.699	0.916									
PE	0.728	0.638	0.731	0.845								
EE	0.496	0.505	0.571	0.603	0.761							
SI	0.274	0.401	0.362	0.357	0.339	0.828						
FC	0.213	0.108	0.228	0.200	0.302	0.157	0.904					
HM	0.237	0.338	0.252	0.300	0.382	0.559	0.156	0.831				
PO	0.305	0.386	0.346	0.424	0.287	0.273	0.276	0.205	0.860			
HT	0.305	0.309	0.290	0.401	0.308	0.200	0.393	0.299	0.725	0.914		
DI	0.495	0.497	0.689	0.632	0.524	0.351	0.242	0.418	0.270	0.354	0.895	
DU	0.502	0.645	0.655	0.613	0.543	0.388	0.215	0.306	0.312	0.263	0.586	1.000

The diagonal value is the square root of the average variance extracted (AVE); the lower triangle is the correlation coefficient of the corresponding variable.

**Table 6:** Heterotrait–monotrait ratio (HTMT).

	TAS	TEC	TTF	PE	EE	SI	FC	HM	PO	HT	DI	DU
TAS	–											
TEC	0.710											
TTF	0.720	0.756										
PE	0.822	0.706	0.826									
EE	0.555	0.554	0.646	0.688								
SI	0.334	0.481	0.439	0.438	0.420							
FC	0.228	0.115	0.245	0.218	0.353	0.184						
HM	0.278	0.397	0.302	0.362	0.482	0.720	0.179					
PO	0.354	0.445	0.400	0.500	0.350	0.352	0.309	0.247				
HT	0.338	0.336	0.322	0.453	0.367	0.243	0.425	0.349	0.843			
DI	0.543	0.531	0.759	0.711	0.593	0.419	0.267	0.504	0.301	0.389		
DU	0.527	0.665	0.688	0.658	0.572	0.448	0.217	0.351	0.343	0.278	0.615	–

### Structural Model Analysis

After the reliability and validity of the measurement model were tested by confirmatory factor analysis, the structure of the model was analyzed further. The PLS algorithm was used to test the fitness of explanatory variables to the prediction of outcome variables, and 5,000 samples were selected by the bootstrap resampling method to calculate the parameters and evaluate the significance of model coefficients (Hair et al., 2011). In the PLS analysis, R^2^ is the primary index used to evaluate the explanatory effectiveness of the model. Chin et al. (2008) divided endogenous variables into three levels according to R^2^, namely, substantial, moderate, and weak, with index values of 0.67, 0.33, and 0.19, respectively. Therefore, the three indicators of 0.556, 0.578, and 0.344 identified in this study are moderate. On this basis, to further determine the stability and fitness of the model, the predictive sample reuse technique (Q^2^) was used, where Q^2^ greater than 0 indicates that the model has predictive relevance (Chin et al., 2008). The Q^2^ value obtained in this study was between 0.331 and 0.437, indicating that the model has cross validity.

The test results for the structural equation model are shown in Table 7. TAS had a significant positive effect on TTF; therefore, *H1a* was supported. TEC had a significant positive effect on TTF; therefore, *H1b* was supported. TTF had a significant positive effect on use intentions for digital museums; therefore, *H1c* was supported. PE had a significant positive effect on use intentions for digital museums; therefore, *H2a* was supported. HM had a significant positive effect on use intentions for digital museums; therefore, *H3a* was supported. PO had a significant negative effect on use intentions for digital museums; therefore, *H3b* was supported. HT had a significant positive effect on use intentions for digital museums; therefore, *H3c* was supported. Use intentions for digital museums had a significant positive effect on use behavior for digital museums; therefore, *H4* was supported. However, EE, SI, and FC had no significant effect on use intentions for digital museums; therefore, *H2b*, *H2c*, and *H2d* were not supported.

**Table 7:** Regression weights among the proposed relationships.

	Original sample (O)	Standard deviation (STDEV)	*T* statistics	*P* values
H1a TAS → TTF	0.345	0.069	4.999	0.000
H1b TEC → TTF	0.472	0.075	6.277	0.000
H1c TTF → DI	0.471	0.072	6.525	0.000
H2a PE → DI	0.196	0.080	2.458	0.014
H2b EE → DI	0.048	0.063	0.755	0.451
H2c SI → DI	−0.017	0.061	0.284	0.777
H2d FC → DI	0.028	0.059	0.483	0.629
H3a HM → DI	0.210	0.059	3.555	0.000
H3b PO → DI	−0.158	0.073	2.172	0.030
H3c HT → DI	0.168	0.079	2.110	0.035
H4 DI → DU	0.586	0.043	13.676	0.000

**Figure 4:**
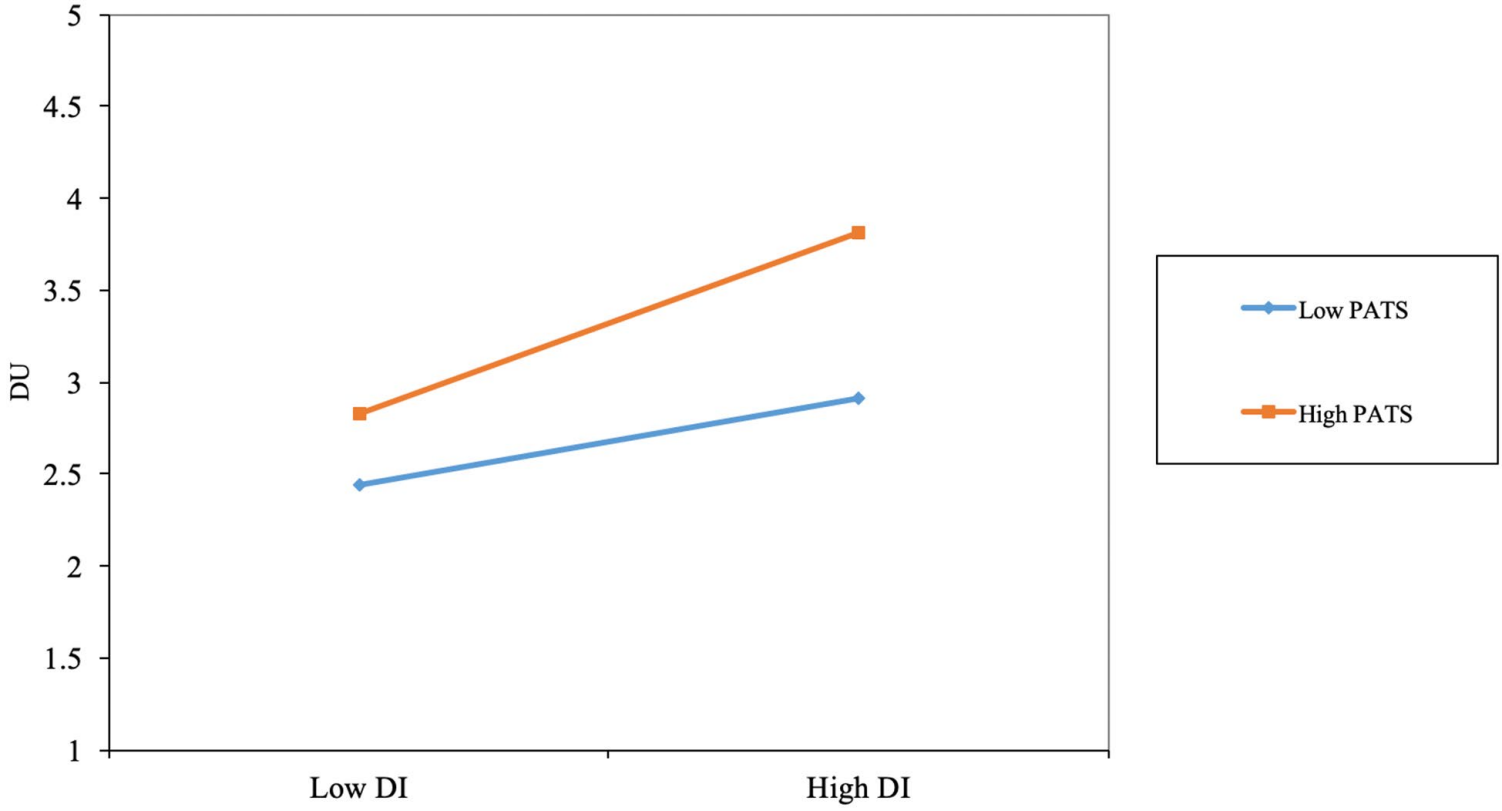
The slope test results.

The moderating effect of body distance on *H4* was analyzed (Table 8). Using the goodness-of-fit (GoF) index proposed by Tenenhaus et al. (2005), the GoF value in this study is 0.432, which is higher than the standard GoF value of 0.36 proposed by Wetzels et al. (2009), indicating good fit. The *p* values are used as the criterion to evaluate whether the moderating effect is significant. The results show that *H5* (*p* < 0.05) is significant. That is, the effect of use intentions on use behavior is moderated by travel anxiety. When travel anxiety is relatively high, use intentions have a greater effect on use behavior for digital museums (Figure 4).

**Table 8:** Moderating effects among the proposed relationships.

	Original sample (O)	Standard deviation (STDEV)	*T* statistics	*P* values
DI → DU	0.363	0.082	4.405	0.000
PAT → DU	0.321	0.077	4.150	0.000
DI* PAT → DU	0.129	0.047	2.744	0.006

## Conclusion, Limitations, and Future Research

### Theoretical Conclusions

This study examines visitors’ use intentions and use behavior for digital museums by integrating the new UTAUT (UTAUT2) and new TTF (TTF under social distancing) models to explore how the supply side and user factors affect the willingness of potential museum visitors to use the digital platform, and introduces the PATS model to reveal how pandemic anxiety can limit travel behavior and promote the transformation of use intentions into use behavior more effectively for digital museums.

The previous studies analyzed digital tools in tourism as an auxiliary tool in simplifying the payment or enhancing tourism experience. Such as, Skard et al. (2021) revealed the effect of digital experience on tourism intentions; Shin and Jeong (2022) explored the virtual trip as a driver evoking future intentions to visit. However, this study does not take digital tool as the antecedent or auxiliary function before or during the on-site experiences as in previous studies, but as a substitute for on-site visits during the pandemic. In addition, we discussed the production process of use intentions and its transformation into use behavior for digital museums.

In our analysis of use intentions, we found that except for PE, three of the four influential factors (i.e., EE, SI, and FC) in the traditional UTAUT model had no significant effect on use intentions for digital museums in this study. This finding differs from the conclusions of earlier studies (Martín and Herrero, 2012; García-Milon et al., 2021). Whether the task is simple or the equipment is easy to use will not affect use intentions. This result shows that as people become increasingly familiar with digital products, they take for granted their simple and good operation. At the same time, social influence will not significantly affect use intentions and behavior for digital museums, which is different from earlier studies (e.g., Šumak and Šorgo, 2016), however, consistent with the recent study (Erjavec and Manfreda, 2022), that is, SI does not have a significant role in behavioral intentions to adopt online shopping. It shows that, as a product based on personal smartphones, willingness to use digital museums is gaining more and more privacy characteristics and the choices are more personalized.

The UTAUT2 model shows that two influential factors, HM and HT, had significant positive effects on use intentions, which shows that use intentions for digital museums can be based on personal factors to a greater extent, including personal experiences, behavior, and interests. From the perspective of the UTAUT2 model, the satisfaction of personal needs, rather than social expectations, has become the main variable affecting use intentions for digital museums. Studies found that VR will lead to isolation in the future (Merkx and Nawijn, 2021) and may reduce actual travel intention (Li and Chen, 2019). Furthermore, this study found that during the pandemic, people were excluded from the social environment for longer and must endure social and physiological isolation, which means that the production of their use intentions or decisions is less influenced by social peers. These states of isolation and digitization are constantly promoting each other, which will make people become increasingly accustomed to physical and psychological isolation. Because the pandemic hinders offline consumption, people have the need for psychological compensation. Wang and Xia (2021) pointed out the “revenge travel phenomenon,” this study revealed the “revenge consumption” before people could go on a real travel. The more people want to consume, the more willing they are to participate in consuming digital museum products. Therefore, there are also opportunities for the promotion of cultural products during the pandemic.

From the perspective of the TTF model, the characteristics of tasks and technologies have changed in the context of the pandemic, namely, TAS and TEC promote people’s use intentions for digital museums. The need to avoid the risk generated by the crowding (Yin et al., 2020) makes people more willing to use digital museum products.

Furthermore, this study introduced PATS as a moderating variable to analyze the transformation from use intentions to use behavior. Gössling et al. (2020) analyzed the negative psychological results of tourists during the pandemic and restrictions on travel behavior. This study goes further and analyzes how this fear of travel promotes the transformation of use intentions into actual use behavior.

We found that when people have higher travel anxiety, their willingness to use digital museums can be transformed into use behavior more easily. When people have lower travel anxiety, they have more confidence in travel. Even if they have the willingness to use digital museums, their use intentions may not be transformed into use behavior for digital museums.

### Managerial Implications

Based on our theoretical analysis, destinations must pay attention to the satisfaction of users’ personal needs from digital museums rather than the satisfaction of their social expectations. Hence, we produce the following management recommendations.

First, the positive effect of the TTF under social distance model and the performance expectations of the UTAUT2 model on use intentions show that people hope that the digital museum experience can not only meet their basic needs, but also obtain the experience faster and more efficiently. The loss of digital product users often depends on a few seconds of page loading. Therefore, waiting for pages to be converted for smartphones will make these users quickly lose patience. Hence, digital museums should improve their perceived efficiency for users with timely updates of content and improve their technological fluency.

Second, based on the positive effect of HM on use intentions, digital products must create a fun, enjoyable, and entertaining environment instead of delivering a serious history lesson. Especially for museums like the Palace Museum in particular, which is based on the Forbidden City’s long history and royal culture, innovation is particularly important to attract consumers in the digital age. It is necessary to ensure high-quality aesthetic design, construct participatory game activities, introduces virtual communication, and even introduce role-playing among other modes, so that the participants are not only holding their smartphone to “see” museum exhibits and learn about history but can engage in lively role-playing activities in an interesting digital historical world.

Third, a “personal relationship” with users could be beneficial for both digital museums and their users. The digital platform not only designs virtual museums but is also included in a handheld companion that is integrated with the users’ daily life. For example, digital museums could launch regular participation activities and communications, establish a “personal platform” relationship or a communication network between platform users, establish long-term relationships between themselves and users, and provide people with a digital community and co-creation experience (Lam et al., 2020).

Fourth, digital museums can meet their users’ need to maintain social distance during the pandemic (Im et al., 2021) and facilitate museum experiences while safely staying at home. In this way, the pandemic becomes an intensive driver for the development of digital museums. To utilize the driving force, museums should build digital experiences that differ from on-site experiences. In this way, they can give full play to the new functions that can be realized by the digital platform, such as providing 3D videos so that people can watch cultural relics at a close distance and allow them to experience protected buildings that cannot otherwise be accessed on-site through virtual “access.” These activities would improve the vividness of users’ knowledge and participation through their pleasant experiences, and emotional connections. Digital museums could then improve their brand image by including personality and character in their digital experiences.

Finally, in the context of the pandemic, digitization enables people in our modern society to meet their daily needs without leaving home, which makes them more physically isolated. Digital museums have the responsibility and ability to improve the social condition of their users by increasing their interactive and communicative functions. This would promote their users’ emotional connections on digitalized platforms and enhance their pleasure while providing historical knowledge. Therefore, digital museums not only explore and develop new markets, but also have great social and ethical significance.

### Limitations and Future Research

Digital museums bring convenience to people with limited travel abilities or people who are afraid of the increasing risks in the travel environment (Yen et al., 2021). However, for those who are not good at using electronic products, large-scale digital constructions, especially in the development of digital museums that replace on-site facilities with digital facilities, may cause more inequality in accessibility, such as for the seniors or people with visual impairments. These groups of people often have high levels of travel anxiety and great potential demand for user-friendly digital products. Museums must confront this contradiction and explore how to provide useful and valuable digital products for these groups, which is also a very valuable research direction for the future.

## Data Availability Statement

The raw data supporting the conclusions of this article will be made available by the authors, without undue reservation.

## Author Contributions

JS: methodology, data curation, formal analysis, writing, and funding acquisition. YG: conceptualization and supervision. All authors contributed to the article and approved the submitted version.

## Funding

This research was supported by the National Natural Science Foundation of China (42101225 and 72074053), Humanities and Social Sciences Youth Foundation, Ministry of Education of the People’s Republic of China (21YJCZH140), and China Postdoctoral Science Foundation (2021 M690652).

## Conflict of Interest

The authors declare that the research was conducted in the absence of any commercial or financial relationships that could be construed as a potential conflict of interest.

## Publisher’s Note

All claims expressed in this article are solely those of the authors and do not necessarily represent those of their affiliated organizations, or those of the publisher, the editors and the reviewers. Any product that may be evaluated in this article, or claim that may be made by its manufacturer, is not guaranteed or endorsed by the publisher.
